# Effectiveness of the Pfizer-BioNTech COVID-19 Vaccine Among Residents of Two Skilled Nursing Facilities Experiencing COVID-19 Outbreaks — Connecticut, December 2020–February 2021

**DOI:** 10.15585/mmwr.mm7011e3

**Published:** 2021-03-19

**Authors:** Amadea Britton, Kara M. Jacobs Slifka, Chris Edens, Srinivas Acharya Nanduri, Stephen M. Bart, Nong Shang, Adora Harizaj, Jillian Armstrong, Kerui Xu, Hanna Y. Ehrlich, Elizabeth Soda, Gordana Derado, Jennifer R. Verani, Stephanie J. Schrag, John A. Jernigan, Vivian H. Leung, Sunil Parikh

**Affiliations:** ^1^CDC COVID-19 Emergency Response Team; ^2^Epidemic Intelligence Service, CDC; ^3^Connecticut Department of Public Health, Hartford, Connecticut; ^4^Yale School of Public Health, New Haven, Connecticut.

Residents of long-term care facilities (LTCFs), particularly those in skilled nursing facilities (SNFs), have experienced disproportionately high levels of COVID-19–associated morbidity and mortality and were prioritized for early COVID-19 vaccination (*1*,*2*). However, this group was not included in COVID-19 vaccine clinical trials, and limited postauthorization vaccine effectiveness (VE) data are available for this critical population (*3*). It is not known how well COVID-19 vaccines protect SNF residents, who typically are more medically frail, are older, and have more underlying medical conditions than the general population (*1*). In addition, immunogenicity of the Pfizer-BioNTech vaccine was found to be lower in adults aged 65–85 years than in younger adults (*4*). Through the CDC Pharmacy Partnership for Long-Term Care Program, SNF residents and staff members in Connecticut began receiving the Pfizer-BioNTech COVID-19 vaccine on December 18, 2020 (*5*). Administration of the vaccine was conducted during several on-site pharmacy clinics. In late January 2021, the Connecticut Department of Public Health (CT DPH) identified two SNFs experiencing COVID-19 outbreaks among residents and staff members that occurred after each facility’s first vaccination clinic. CT DPH, in partnership with CDC, performed electronic chart review in these facilities to obtain information on resident vaccination status and infection with SARS-CoV-2, the virus that causes COVID-19. Partial vaccination, defined as the period from >14 days after the first dose through 7 days after the second dose, had an estimated effectiveness of 63% (95% confidence interval [CI] = 33%–79%) against SARS-CoV-2 infection (regardless of symptoms) among residents within these SNFs. This is similar to estimated effectiveness for a single dose of the Pfizer-BioNTech COVID-19 vaccine in adults across a range of age groups in noncongregate settings (*6*) and suggests that to optimize vaccine impact among this population, high coverage with the complete 2-dose series should be recommended for SNF residents and staff members. 

After identification of the first infected SNF resident or staff member through weekly surveillance testing, expanded facility-wide outbreak SARS-CoV-2 testing was performed frequently for residents and staff members at both facilities in accordance with CDC and CT DPH guidelines (*7*). All residents who had not received a positive test result in the preceding 90 days, regardless of symptoms, received a once-weekly (facility A) or twice-weekly (facility B) polymerase chain reaction (PCR) test. Staff members were also tested regularly (once-weekly antigen and once-weekly PCR test at facility A, and once-weekly PCR test at facility B). At both facilities, supplementary antigen testing was performed immediately for any resident or staff member who developed COVID-19 symptoms and for residents who had known COVID-19 exposures.

A retrospective cohort investigation using data from electronic medical record chart abstraction was conducted to assess vaccine effectiveness. This activity was reviewed by CDC and was conducted consistent with applicable federal law and CDC policy.^§^ The investigation period started on the date of each SNF’s first vaccination clinic (December 29, 2020 for facility A and December 21, 2020 for facility B) and ended on February 9, 2021 and February 12, 2021, respectively. Residents were included if they were admitted at either facility during one or more rounds of facility-wide SARS-CoV-2 testing during the week before or any time after their facility’s first vaccination clinic. Data on residents were abstracted starting on the date of their SNF’s first vaccination clinic or their admission into the facility, whichever occurred later. Electronic medical record data included demographic characteristics, facility admission and discharge dates, vaccination dates, symptoms of COVID-19 occurring within 7 days before or 14 days after a positive test result, presence of underlying medical conditions associated with potential increased risk for severe COVID-19 illness,^¶^ and measures of outcome, including hospitalization and death. SARS-CoV-2 test dates, test types, and results were also obtained from the electronic medical record.

A case was defined as any positive PCR- or antigen-based SARS-CoV-2 test result during the investigation period in a resident meeting the cohort inclusion criteria. Case date was defined as either the date of symptom onset or positive SARS-CoV-2 test result, whichever occurred earlier. Positive SARS-CoV-2 test results received before the investigation period were identified for each resident using the Connecticut Electronic Disease Surveillance System.

Person-time began on the date of the facility’s first vaccination clinic or the date the resident was admitted, whichever occurred later. Residents stopped contributing person-time to the investigation on the case date, the final facility discharge date or date of death if applicable, or the final day of the investigation period, whichever occurred earlier. Resident person-time was categorized as 1) unvaccinated (days from cohort entry until receipt of first vaccine dose), 2) time before first vaccine dose effect (day 0 [date of vaccination] through day 14 after first dose), 3) partially vaccinated (>day 14 after first dose through day 7 after second dose), or 4) fully vaccinated (>7 days after second dose).

Assuming a common VE against SARS-CoV-2 infection at both facilities, a Cox proportional hazards model with baseline hazard rates stratified by facility was applied to estimate the VE, with VE = 100% × (1−hazard ratio); 95% CIs were calculated using robust CI methods.** Use of a time-to-event analysis was necessary to adjust for expected heterogeneity in risk for infection across the investigation period attributable to underlying outbreak dynamics. Kaplan-Meier curves of SARS-CoV-2 infection were constructed to visualize the cumulative infection-free proportion of residents; 95% CIs were calculated using Greenwood’s method.^††^ Sensitivity analyses were conducted with exclusion of residents with past confirmed SARS-CoV-2 infection and using two alternative endpoints for partial vaccination (ending on second dose +0 days and second dose +14 days). The time before first dose vaccine effect was excluded from the analysis, because immune status could not be clearly categorized. Small sample sizes precluded separate analyses of VE against symptomatic or severe disease. R statistical software (version 4.0.2; The R Foundation) was used to conduct all analyses.

A total of 463 residents were enrolled, including 142 (31%) from facility A and 321 (69%) from facility B. Demographic characteristics such as age and race were similar in residents at each facility (although ethnicity could not be reported because ethnicity data were missing for 30% of residents); prevalences of underlying conditions that increase the risk for severe COVID-19 illness were also similar in residents at each facility (Table). The median number of high-risk conditions per resident was three; five (1.1%) residents had no underlying high-risk conditions. Among the 463 residents, 115 (24.8%) had confirmed SARS-CoV-2 infection before the investigation period; two of 34 (6%) residents at facility A and 68 of 81 (84%) residents at facility B with past confirmed SARS-CoV-2 infection had a positive test result ≤3 months prior to investigation start.

**TABLE:** Demographic characteristics, COVID-19 vaccination status, and SARS-CoV-2 infection, symptom, and outcome information among residents of two skilled nursing facilities — Connecticut, December 21, 2020–February 12, 2021

Characteristic	No. (%) of residents*			
	Total	Facility A	Facility B	p-value^†,§^
	(N = 463)	(n = 142)	(n = 321)	
**Sex**				
Female	**294 (63.5)**	82 (57.8)	212 (66.0)	0.09
Male	**169 (36.5)**	60 (42.3)	109 (34.0)	
**Age group, yrs**				
<60	**23 (5.0)**	18 (12.7)	5 (1.6)	<0.001
60–64	**19 (4.1)**	12 (8.5)	7 (2.2)	
65–69	**34 (7.3)**	16 (11.3)	18 (5.6)	
70–74	**46 (9.9)**	14 (9.9)	32 (10.0)	
75–79	**56 (12.1)**	17 (12.0)	39 (12.2)	
80–84	**54 (11.7)**	15 (10.6)	39 (12.2)	
≥85	**231 (49.9)**	50 (35.2)	181 (56.4)	
**Race^¶^**				
American Indian/Alaska Native	**1 (0.2)**	0 (0.0)	1 (0.3)	0.57^§^
Asian	**5 (1.1)**	1 (0.7)	4 (1.3)	
Black	**16 (3.5)**	5 (3.5)	11 (3.4)	
Native Hawaiian/Pacific Islander	**1 (0.2)**	0 (0.0)	1 (0.3)	
White	**428 (92.4)**	135 (95.1)	293 (91.3)	
Unknown	**12 (2.6)**	1 (0.7)	11 (3.4)	
High-risk medical conditions**				
Obesity	**44 (9.5)**	16 (11.3)	28 (8.7)	0.39
Chronic kidney disease	**92 (19.9)**	32 (22.5)	60 (18.7)	0.34
End-stage renal disease requiring dialysis	**3 (0.7)**	2 (1.4)	1 (0.3)	0.22^§^
Diabetes mellitus (type I or II)	**131 (28.3)**	51 (35.9)	80 (24.9)	0.02
Cancer (not in remission)	**28 (6.1)**	9 (6.3)	19 (5.9)	0.86
Autoimmune disease	**33 (7.1)**	13 (9.2)	20 (6.2)	0.26
Chronic heart or cardiovascular disease	**186 (40.2)**	55 (38.7)	131 (40.8)	0.67
Hypertension	**352 (76.0)**	103 (72.5)	249 (77.6)	0.24
COPD/Sleep apnea/Other chronic respiratory condition	**94 (20.3)**	34 (23.9)	60 (18.7)	0.20
Immunocompromising conditions^††^	**9 (1.9)**	4 (2.8)	5 (1.6)	0.47^§^
Neurologic/Neurodevelopmental disorders^§§^	**346 (74.7)**	105 (73.9)	241 (75.1)	0.80
Other chronic diseases	**66 (14.3)**	7 (4.9)	59 (18.4)	0.001
None of these conditions	**5 (1.1)**	1 (0.7)	4 (1.2)	0.10^§^
**History of past COVID-19**				
Yes	**115 (24.8)**	34 (23.9)	81 (25.2)	0.76
>3 months before investigation start	**45 (9.7)**	32 (22.5)	13 (4.0)	<0.001
≤3 months before investigation start	**70 (15.1)**	2 (1.4)	68 (21.2)	
**Vaccination coverage among all residents^¶¶^**				
None	**87 (18.8)**	32 (22.5)	55 (17.1)	0.09
1 dose only	**72 (15.6)**	27 (19.0)	45 (14.0)	
2 doses	**304 (65.7)**	83 (58.5)	221 (68.8)	
**Interval between vaccine doses**				
Days between doses 1 and 2, median (range)	**21 (21–42)**	21 (21–42)	21 (21–32)	N/A
**Cases**				
All cases	**97 (21.0)**	40 (28.2)	57 (17.8)	0.01
Symptomatic, no. (% of cases)	**86 (88.7)**	33 (82.5)	53 (93.0)	0.19^§^
**Reported symptoms, no. (% of cases)**				
None	**11 (11.3)**	7 (17.5)	4 (7.0)	0.19^§^
Fever and chills	**24 (24.7)**	5 (12.5)	19 (33.3)	0.02
Cough	**63 (65.0)**	21 (52.5)	42 (73.7)	0.03
Shortness of breath/Difficulty breathing	**18 (18.6)**	8 (20.0)	10 (17.5)	0.76
Myalgias	**7 (7.2)**	0 (0.0)	7 (12.3)	0.04^§^
Headaches	**3 (3.1)**	2 (5.0)	1 (1.8)	0.57^§^
Sore throat	**5 (5.2)**	0 (0.0)	5 (8.8)	0.08^§^
New loss of taste or smell	**1 (1.0)**	0 (0.0)	1 (1.8)	N/A
Congestion/Rhinorrhea	**16 (16.5)**	6 (15.0)	10 (17.5)	0.74
Abdominal pain	**3 (3.1)**	2 (5.0)	1 (1.8)	0.57^§^
Nausea/Vomiting	**12 (12.4)**	2 (5.0)	10 (17.5)	0.11^§^
Diarrhea	**6 (6.2)**	1 (2.5)	5 (8.8)	0.40^§^
Confusion/Altered mental status	**21 (21.7)**	11 (27.5)	10 (17.5)	0.24
Other***	**65 (67.0)**	26 (65.0)	39 (68.4)	0.72
**Vaccination status on case date, no. (% of cases)**				
Unvaccinated	**39 (40.2)**	15 (37.5)	24 (42.1)	0.16^§^
Before dose 1 effect (day 0 through day 14 after dose 1)	**26 (26.8)**	15 (37.5)	11 (19.3)	
Partially vaccinated (>day 14 after dose 1 through day 7 after dose 2)	**25 (25.8)**	9 (22.5)	16 (28.1)	
Fully vaccinated (>7 days after dose 2)	**7 (7.2)**	1 (2.5)	6 (10.5)	
**Outcomes,^†††^ no. (% of cases)**				
Hospitalization	**15 (15.5)**	4 (10.0)	11 (19.3)	0.21
Vital status dead or unknown				
Death from COVID-19	**17 (17.5)**	7 (17.5)	10 (17.5)	0.55^§^
Death after diagnosis (no cause specified)	**4 (4.1)**	1 (2.5)	3 (5.3)	
Vital status unknown	**3 (3.1)**	0 (0.0)	3 (5.3)	

**Abbreviations:** COPD = chronic obstructive pulmonary disease; N/A = not applicable.

* Percentages might not sum to 100% because of rounding.

^†^ P-values for the comparisons between facilities apply Pearson’s chi-square test for independence unless marked. For mutually exclusive categories of a characteristic a single p-value is reported. For characteristics for which more than one category might be true for a resident (e.g., symptoms), individual p-values are reported for each category.

^§^ In cases with cell counts <5, Fisher’s exact test was used to calculate the p-value.

^¶^ Ethnicity is not reported because data were missing for 30% of residents.

** Conditions associated with potential increased risk for severe COVID-19 illness per CDC guidelines.

^††^ HIV coinfection (not virally suppressed), chemotherapy within past 12 months, solid-organ or bone marrow transplant, long-term steroid use (20 mg per day for >1 month), taking immunosuppressants, or taking tumor necrosis factor-alpha inhibitors.

^§§^ Examples include seizure disorders such as epilepsy, Alzheimer disease, dementia, traumatic brain injuries, and stroke.

^¶¶^ Vaccination is reported as the percentage of all residents included in the investigation that received no dose, 1 dose, or 2 doses of Pfizer-BioNTech COVID-19 vaccine by the date of their discharge from the facility or the end of the investigation if they were still admitted to the facility. Absolute coverage in the facility changed daily because of changes in census.

*** Other symptoms included lethargy, fatigue, generalized weakness, malaise, decreased appetite or loss of appetite, and agitation.

^†††^ Case outcomes include minimum number of confirmed COVID-19–related hospitalizations and COVID-19 deaths confirmed by the Office of the Chief Medical Examiner. Hospitalizations and deaths that occurred after the investigation period were not ascertained.

During the investigation period, 97 cases of SARS-CoV-2 infection occurred, including 40 (41%) at facility A and 57 (59%) at facility B (Figure 1). Including nonspecific symptoms such as malaise, lethargy, and decreased appetite, at least one COVID-19 symptom was reported in 86 (88.7%) cases.^§§^ By the date of discharge or the last day of the investigation, 304 residents (65.7%) had received 2 vaccine doses, 72 (15.6%) had received 1 dose only, and 87 (18.8%) had not received any doses. A total of 16,969 person-days were observed during the investigation period, with 39 cases occurring during 3,573 days categorized as unvaccinated person-time, 26 cases during 4,588 days of person-time before first vaccine dose effect, 25 cases during 4,147 days of partially vaccinated person-time, and seven cases during 4,661 days of fully vaccinated person-time.

**FIGURE 1:**
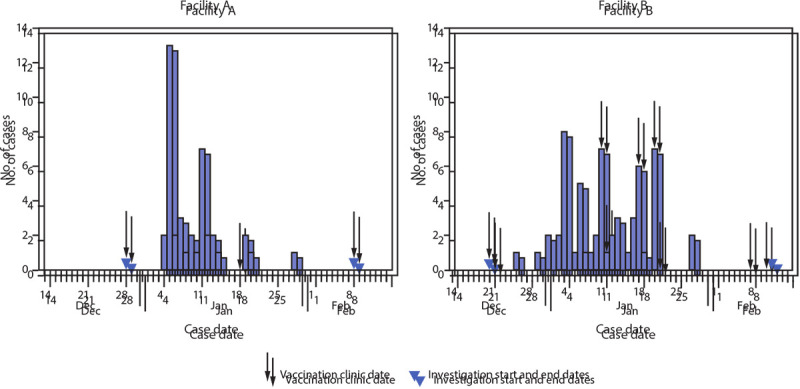
New SARS-CoV-2 cases* among residents of two skilled nursing facilities, by case date^†^ — Connecticut, December 21, 2020–February 12, 2021^§^ * Any positive SARS-CoV-2 polymerase chain reaction or antigen test result. ^†^ Symptom onset date or positive test result date, whichever occurred earlier. ^§^ Investigation period was December 29, 2020–February 9, 2021 for facility A and December 21, 2020–February 12, 2021 for facility B.

Estimated effectiveness of partial vaccination in preventing SARS-CoV-2 infection was 63% (95% CI = 33%–79%) and was similar when residents with past SARS-CoV-2 were excluded (VE = 60%, 95% CI = 30%–77%). VE estimates were also similar in both partial vaccination endpoint sensitivity analyses (second dose +0 days VE = 66%, 95% CI = 29%–83%; second dose +14 days VE = 60%, 95% CI = 33%–77%). As a result of the course of the outbreaks at both facilities, most cases occurred toward the start of the investigation period (Figure 2), and because the cohort began at the first vaccination clinic, most of the unvaccinated person-time also occurred toward the start of the investigation period. Thus, once residents became fully vaccinated (second dose +7 days) toward the end of the investigation period, there were insufficient new cases and remaining person-time in the unvaccinated group to serve as a comparator for estimation of full 2-dose VE.

**FIGURE 2:**
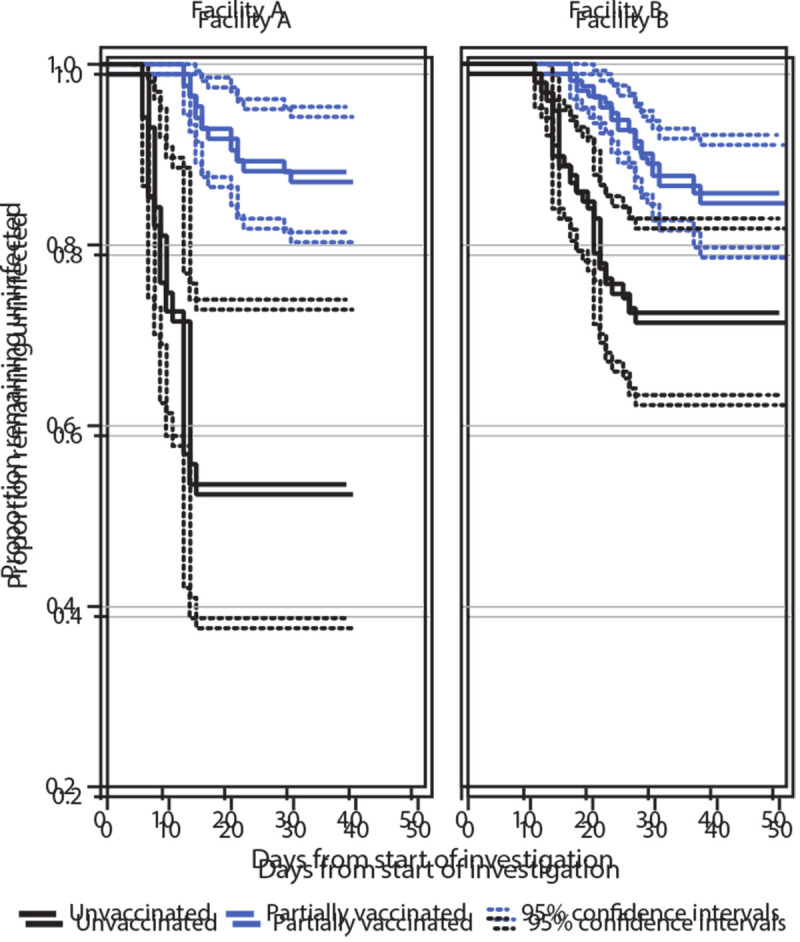
Proportion of skilled nursing facility residents who remained uninfected with SARS-CoV-2 during the investigation period,* by COVID-19 vaccination status^†^ and facility — Connecticut, December 21, 2020–February 12, 2021 * Investigation period was December 29, 2020–February 9, 2021 for facility A and December 21, 2020–February 12, 2021 for facility B. ^†^ Vaccination status is classified as unvaccinated or partially vaccinated. Partially vaccinated refers to the time from day 14 after first dose of Pfizer-BioNTech COVID-19 vaccine through day 7 after the second dose. Greenwood’s method was used to estimate confidence intervals around the Kaplan-Meier estimator.

## Discussion

Partial vaccination with the Pfizer-BioNTech COVID-19 vaccine was 63% effective in preventing new SARS-CoV-2 infections in SNF residents, a disproportionately affected population excluded from initial preauthorization vaccine clinical trials. Even during a large disease outbreak in a long-term care setting, the Pfizer-BioNTech vaccine provided protection against SARS-CoV-2 infection, including in older adults aged ≥65 years with a high prevalence of underlying medical conditions. The findings in this report are comparable to other first-dose vaccine efficacy and effectiveness estimates for the Pfizer-BioNTech vaccine for the broader adult population in noncongregate settings. In the phase 3 clinical trial, efficacy during the interval between first and second doses was estimated at 52% (95% CI = 30%–68%) (*8*). In a recent study of the Pfizer-BioNTech vaccine in Israel, effectiveness against PCR-confirmed infection in the general adult population during days 14–20 and 21–27 after the first dose was 46% (95% CI = 40%–51%) and 60% (95% CI = 53%–66%, respectively) (*6*). Effectiveness was somewhat lower during days 14–20 and 21–27 among persons aged ≥70 years (22%; 95% CI = −9%–44% and 50%; 95% CI = 19%–72%, respectively) and among those with three or more underlying medical conditions (37%; 95% CI = 12%–55% and 37%; 95% CI = −1%–62%) (*6*).

In this investigation, nearly 25% of residents had confirmed past SARS-CoV-2 infection. Serologic studies have indicated that preexisting immunity might strengthen the response to a single dose of COVID-19 vaccine (*9*). A sensitivity analysis excluding person-time contributed by residents with confirmed past infections did not substantially alter VE estimates for residents receiving the first vaccine dose. Among residents in this investigation with past confirmed SARS-CoV-2 infection, first-dose vaccination rates were >90%, and only one reinfection was documented, limiting the ability to determine the impact of past infection.

The findings in this report are subject to at least seven limitations. First, because there were no clear factors that would differentially affect the risk for infection among residents within either facility, such as units with higher attack rates or different infection prevention practices, each observation in the model was treated as independent. If risk was not independent, this could have biased the VE estimates. Second, 2-dose VE estimates were not possible because unvaccinated cases and person-time after second-dose vaccination clinics were insufficient. Third, small sample sizes did not allow for analyses of secondary endpoints, such as effectiveness against symptomatic illness, hospitalization, or death. Fourth, although there was no change in guidance around outbreak control measures such as cohorting and other infection prevention and control strategies concurrent with vaccine introduction, had these measures been implemented differently for vaccinated and unvaccinated residents, VE estimates could have been biased. Fifth, racial minority groups were underrepresented in this investigation compared with the general population of older adults, and ethnicity data were missing for approximately one third of residents, which might affect generalizability to other SNF populations. Sixth, although excluding person-time from residents with known past confirmed SARS-CoV-2 infection did not influence VE estimates in this analysis, there could have been residents with unknown past infection who could still have acted as a source of potential bias. Finally, unrecognized underlying differences between vaccinated and unvaccinated residents might have confounded the effectiveness estimates. Strengths of the investigation include accurate collection of vaccination data through direct abstraction from resident electronic medical records and active ascertainment of SARS-CoV-2 infection through frequent, facility-wide resident testing.

Findings from this retrospective cohort analysis demonstrate that partial vaccination with the Pfizer-BioNTech COVID-19 vaccine was associated with a significant reduction in the risk for SARS-CoV-2 infection among SNF residents. These results, coupled with the findings from a previous study among comparable older adult populations in Israel that reported more robust protection after the second dose (*6*), suggest that complete 2-dose vaccination is an important strategy for preventing COVID-19 in this disproportionately affected population. Further study of this population should continue as larger sample sizes become available. LTCFs and jurisdictions should actively ensure that they have plans in place for continued allocation and administration of COVID-19 vaccines to residents and staff members (*10*).

**Summary**

**What is already known about this topic?**

Skilled nursing facility (SNF) residents, generally older and with more underlying medical conditions than community-dwelling adults, were not included in COVID-19 vaccine clinical trials. Little is known about COVID-19 vaccine effectiveness in SNF residents.

**What is added by this report?**

A retrospective cohort analysis in two Connecticut SNFs found partial vaccination with Pfizer-BioNTech COVID-19 vaccine (from >14 days after dose 1 through 7 days after dose 2) to be 63% (95% confidence interval = 33%–79%) effective against SARS-CoV-2 infection.

**What are the implications for public health practice?**

Even with partial vaccination, Pfizer-BioNTech COVID-19 vaccine provides protection to SNF residents. To optimize vaccine impact among this population, high coverage with the complete 2-dose series is recommended.

## Footnotes

* These authors contributed equally as first authors.

† These authors contributed equally as senior authors.

§ This investigation was defined as having met the requirements for public health surveillance as outlined in 45 C.F.R. part 46.102(l)(2), 21 C.F.R. part 56; 42 U.S.C. Sect. 241(d); 5 U.S.C. Sect. 552a; 44 U.S.C. Sect. 3501 et seq.

¶ Conditions based on CDC guidelines identifying conditions associated or potentially associated with risk for severe COVID-19 illness. List of conditions available at https://www.cdc.gov/coronavirus/2019-ncov/need-extra-precautions/people-with-medical-conditions.html.

** Halloran ME, Longini IM Jr, Struchiner CJ. Design and analysis of vaccine studies. Statistics for biology and health. New York, NY: Springer; 2009.

†† Greenwood M. The natural duration of cancer. In: Reports on public health and medical subjects. London, United Kingdom: Her Majesty's Stationery Office; 1926:1–26.

§§ Clinician judgement during chart abstraction was used to distinguish COVID-19 symptoms from those potentially associated with vaccination or other illness. Symptoms had to be new onset within 7 days before or 14 days after a positive test result. Symptom-onset date was available for 80 of 86 cases classified as symptomatic (93%). Among those 80 cases for which symptom-onset date was available, only four (5%) had a symptom-onset date within the 48 hours after receiving a vaccine.
